# Lipoprotein(a) levels and estimated QRISK3 cardiovascular risk in the Randox Health 2025 cohort: a cross-sectional analysis of 14,848 individuals

**DOI:** 10.3389/fcvm.2026.1949855

**Published:** 2026-09-16

**Authors:** Joanne Watt, Mary Jo Kurth, Cormac Rice, Jack Green, John V. Lamont, Laura Mooney, Le Roy Dowey, Peter Fitzgerald, Mark W. Ruddock

**Affiliations:** 1 Randox Laboratories Ltd., Clinical Studies Group, Crumlin, United Kingdom; 2 School of Biomedical Sciences, Ulster University, Coleraine Campus, Coleraine, United Kingdom; 3 Randox Health GB, London, London

**Keywords:** cardiovascular risk prediction, dyslipidaemia, lipoprotein(a), population screening, primary prevention, QRISK3

## Abstract

**Introduction:**

Lipoprotein(a) (Lp(a)) is an independent, genetically-determined cardiovascular risk factor not currently incorporated into standard cardiovascular risk prediction models such as QRISK3. Understanding the relationship between Lp(a) and estimated cardiovascular risk may identify individuals whose risk is not identified by conventional risk calculators.

**Methods:**

Analysis was performed on *n* = 14,848 participants from the Randox Health 2025 dataset. QRISK3 scores were estimated as 10-year cardiovascular risk using demographic, clinical, lifestyle, and medical-history, mapped to QRISK3 definitions. Participants were classified with low (<10%) or elevated (≥10%) estimated 10-year cardiovascular risk. Lp(a) concentrations were categorised into three-group-classification: low risk ≤75 nmol/L, grey zone 76–125 nmol/L, and high risk >125 nmol/L. Associations between Lp(a) and QRISK3 groups were assessed using non-parametric tests.

**Results and Discussion:**

Among *n* = 14,848 participants, *n* = 1,426 (9.6%) had estimated QRISK3 score ≥10%, while *n* = 2,670 (18.0%) had Lp(a) >125 nmol/L. Participants with QRISK3 ≥10% were older (64 vs. 40-years; *p* < 0.001), commonly male (77% vs. 23%; *p* < 0.001), higher systolic blood-pressure (140 vs. 124mmHg; *p* < 0.001), higher BMI (27.3 vs. 25.0 kg/m^2^; *p* < 0.001), and a higher prevalence of diabetes and established cardiovascular risk factors (*p* < 0.001). Elevated Lp(a) concentrations (>125 nmol/L) were more common in individuals with QRISK3 scores ≥10% than those individuals with QRISK3 scores <10% (21% vs. 18%; *p* = 0.002). Among individuals with elevated Lp(a), 89% remained below the treatment threshold of QRISK3 <10%. A total of *n* = 2,371 participants (16%) exhibited elevated Lp(a) levels while remaining within low QRISK3 risk.

Median estimated QRISK3 scores increased incrementally across Lp(a) groups (1.4%, 1.4%, and 1.8%, for low-, intermediate-, and high-Lp(a) groups, respectively; *p* < 0.001), although absolute differences were small.

Elevated Lp(a) levels were common within the Randox Health 2025 cohort and demonstrated limited concordance with estimated QRISK3. Most individuals with high Lp(a) had QRISK3 scores below conventional treatment thresholds, indicating discordance between Lp(a)-based risk and QRISK3-derived 10-year risk classification. These findings suggest elevated Lp(a) identifies a cardiovascular risk not incorporated into QRISK3 supporting consideration of Lp(a) measurement alongside conventional cardiovascular risk assessment. Participants were not excluded based on established cardiovascular disease; therefore, the findings should be interpreted as applying to a real-world health-screening population rather than a strictly defined primary-prevention cohort.

## Key messages

1. Lp(a) concentrations (>125 nmol/L) were present in 18% of the Randox Health 2025 cohort.
2. Only 2% of participants had both elevated Lp(a) levels and QRISK3 ≥10%.
3. Nearly 90% of individuals with elevated Lp(a) levels had QRISK3 scores <10%, demonstrating limited overlap between Lp(a)-based risk identification and QRISK3 classification.
4. Lp(a) appears to capture an aspect of cardiovascular risk that is not fully reflected by traditional QRISK3 risk factors.
5. These findings support the view that Lp(a) and QRISK3 provide complementary rather than interchangeable information for cardiovascular risk assessment.

## Introduction

Cardiovascular disease (CVD) remains the leading cause of mortality worldwide despite major advances in risk-factor management and preventive cardiology (1). Accurate identification of individuals at increased risk is therefore fundamental to effective primary prevention. In the United Kingdom, QRISK3 is the predominant tool used to estimate 10-year cardiovascular risk and inform decisions regarding lipid-lowering therapy (2, 3). The algorithm integrates age, sex, ethnicity, smoking status, blood pressure, body mass index, family history, comorbidities, and medication use, providing a robust estimate of short- to intermediate-term cardiovascular risk. However, QRISK3 does not incorporate lipoprotein(a) (Lp(a)), a genetically inherited and increasingly recognised causal risk factor for atherosclerotic cardiovascular disease and calcific aortic valve disease (4).

Lp(a) differs fundamentally from most traditional cardiovascular risk factors (5). Circulating concentrations are largely genetically determined, remain relatively stable throughout life, and are minimally influenced by lifestyle modification (6). Consequently, individuals with markedly elevated Lp(a) may accumulate substantial lifetime exposure to an atherogenic and pro-inflammatory lipoprotein despite having favourable conventional cardiovascular risk profiles (7). In contrast, QRISK3 estimates the probability of a cardiovascular event occurring within the next 10 years based primarily on age-dependent risk factors (3, 8). This creates the potential for discordance between inherited cardiovascular risk and estimated risk derived from traditional prediction algorithms. As a result, elevated Lp(a) may identify individuals whose future risk is not identified by conventional risk calculators, particularly younger adults who have not yet accumulated sufficient traditional risk factors to cross treatment thresholds (4).

Approximately 15%–25% of the population have elevated Lp(a) concentrations, and contemporary international consensus statements now recommend at least one lifetime measurement of Lp(a) (9). Increasing attention has therefore shifted from whether Lp(a) should be measured to how the result should be interpreted and incorporated into routine cardiovascular risk assessment.

A particular area of uncertainty concerns individuals with Lp(a) concentrations that are neither clearly low nor markedly elevated. This intermediate range, often referred to as the “grey zone” (76–125 nmol/L), represents a sizeable proportion of the population and may identify individuals whose risk is higher than suggested by traditional risk scores alone (4, 10). While very high Lp(a) concentrations are increasingly recognised as a risk-enhancing factor, less is known about the relationship between grey-zone Lp(a) concentrations and established cardiovascular risk prediction tools (4).

The aim of this study was therefore to examine the relationship between Lp(a) concentrations and estimated QRISK3 risk in a large contemporary UK health-screening cohort. Specifically, we sought to determine the extent of overlap between elevated Lp(a) levels and QRISK3-derived cardiovascular risk, evaluate the clinical significance of the Lp(a) grey zone, and identify individuals who may benefit from enhanced cardiovascular risk management despite being classified as low risk by QRISK3. By exploring the complementary information provided by these two approaches, we aimed to better define how Lp(a) measurement might be integrated into routine cardiovascular risk stratification.

## Methods

### Study design and population

This was a retrospective cross-sectional analysis of the Randox Health 2025 dataset (UK and Ireland). Participants undergoing health assessment between January 2025 and December 2025 with available Lp(a) measurements and all required variables to estimate QRISK3 risk were included in the study. Individuals were eligible for the study if they were 25–84 years of age and competent to give informed consent. Informed consent was recorded digitally for all study participants through the Randox Health portal; data use was based on individual consent, as provided on 27 March 2026, and all individual data was anonymised and aggregated. A total of *n* = 14,848 individuals were available for the primary analysis.

#### Ethics

The study was reviewed and approved by the Ulster University School of Biomedical Sciences Ethics Filter Committee (Project Number: FCBMS-26-030). Methods were performed in accordance with both the Strengthening the Reporting of Observational Studies in Epidemiology (STROBE) initiative, and the Declaration of Helsinki (1964). It was not possible to involve patients or the public in the design, conduct, reporting, and dissemination plans of this research.

#### Randox health check

Prior to sample collection, individuals completed a health and lifestyle questionnaire that documented lifestyle habits, health concerns, family history of disease(s), and if symptomatic. Anthropometric measurements (weight (kg), height (cm), and BP (mmHg)) were recorded by Randox Health clinical staff. Venous blood was collected and processed (plasma) for biomarker determination, as per manufacturer's instructions.

#### Biomarker analysis

Biomarker levels were measured at Randox Clinical Laboratory Services (RCLS) (Antrim, Northern Ireland, United Kingdom; Accreditation ISO17025) according to manufacturers' instructions. The Randox Lp(a) assay is calibrated in nmol/L and traceable to WHO/IFCC reference material (IFCC SRM 2B)).

#### Estimation of QRISK3

QRISK3 scores were estimated using the published QRISK3 risk prediction algorithm (QRISK3-2018 version) and variables available within the Randox Health database. The reconstructed score incorporated all QRISK3 variables available in the dataset and was intended to provide an estimated 10-year cardiovascular risk rather than a direct replication of the online QRISK3 clinical calculator.

The following variables were available and included in the calculation: age (25–84 years), sex assigned at birth, ethnicity, smoking status, systolic blood pressure (SBP), body mass index (BMI), total cholesterol to HDL-cholesterol ratio, family history of cardiovascular disease, type 1 diabetes, type 2 diabetes, atrial fibrillation, chronic kidney disease, rheumatoid arthritis, systemic lupus erythematosus, migraine, severe mental illness, erectile dysfunction, current statin therapy, steroid treatment, and atypical antipsychotic use.

Two QRISK3 components were unavailable and therefore could not be incorporated into the reconstructed score. First, the Townsend deprivation score was unavailable because postcode-level deprivation data were not collected as part of the health-screening programme. Second, systolic blood pressure variability could not be calculated because repeated blood pressure measurements were not available. These variables were therefore omitted from the reconstruction. Because both variables contribute to the original QRISK3 algorithm, the resulting score should be considered an estimation of QRISK3-derived risk.

Participants were required to have complete data for all variables necessary for score estimation, including age, sex, ethnicity, smoking status, BMI, systolic blood pressure, total cholesterol, and HDL-cholesterol. Individuals with missing data for any of these core variables were excluded from QRISK3 estimation. No imputation of missing values was performed.

Estimated QRISK3 values were subsequently categorised according to contemporary NICE thresholds into low risk (<10%) and elevated risk (≥10%).

#### Lp(a) classification

Three groups were used to classify Lp(a) risk levels; low risk: ≤75 nmol/L, intermediate (“grey zone”): 76–125 nmol/L, and high risk: >125 nmol/L.

### Statistical analysis

All analyses were generated using R, version 4.6.1 (https://www.R-project.org/; Vienna, Austria) (11). Continuous variables are reported as median (interquartile range [IQR]) and compared using Kruskal–Wallis rank sum test. Categorical variables are presented as frequency (%) and compared using Pearson's Chi-squared test. To estimate the strength and direction of the associations, univariable logistic regression models were constructed with Lp(a) risk groupings defined as the dependent variable. Group effects are reported as Odds Ratios (OR) alongside their corresponding 95% Confidence Intervals (CI) and *p*-values. A two-sided *p*-value <0.05 was considered statistically significant.

## Results

### Cohort characteristics

The Randox Health 2025 cohort consisted predominantly of individuals classified as “white or not stated” ethnicity (80% (11,870/14,848)); 3.3% (293/11,870) of individuals in the “white or not stated” category were of unknown ethnicity. Of note, the QRISK3 score is designed for use within a UK population, however, 5.7% (842/14,848) of the cohort was from the Republic of Ireland. Overall, the median age was 42 years, with males representing 55% (8,232/14,848) of the total cohort. Elevated Lp(a) concentrations (>125 nmol/L) were observed in 18.0% (2,670/14,848) of participants, whereas 9.6% (1,426/14,848) had a QRISK3 score ≥10% (Table 1).

**Table 1:** Association between Lp(a) risk categories and the variables used to estimate QRISK3 cardiovascular risk.

Characteristic	Descriptive Statistics				Multinomial Odds Ratios^3^	
	Low Risk *N* = 10,979^a^	Grey Zone *N* = 1,199^a^	High Risk *N* = 2,670^a^	*p*-value^b^	OR (95% CI)^c^	*p*-value^c^
Age (years)	41 (34, 51)	42 (34, 52)	43 (35, 53)	<0.001	1.01 (1.01–1.01)	<0.001
Sex				0.4		
Female	4,875 (44%)	557 (46%)	1,184 (44%)		–	–
Male	6,104 (56%)	642 (54%)	1,486 (56%)		0.98 (0.91–1.05)	0.5
QRISK3 score	1.4 (0.4, 4.2)	1.4 (0.5, 4.6)	1.8 (0.6, 5.1)	<0.001	1.01 (1.00–1.02)	<0.001
Ethnicity						
White, or not stated	8,867 (81%)	854 (71%)	2,149 (80%)		–	–
Indian	462 (4.2%)	79 (6.6%)	98 (3.7%)		1.13 (0.94–1.35)	0.2
Pakistani	192 (1.7%)	25 (2.1%)	30 (1.1%)		0.85 (0.62–1.14)	0.3
Bangladeshi	52 (0.5%)	7 (0.6%)	11 (0.4%)		1.02 (0.58–1.72)	>0.9
Other Asian	170 (1.5%)	15 (1.3%)	21 (0.8%)		0.63 (0.43–0.89)	0.011
Black Caribbean	30 (0.3%)	16 (1.3%)	36 (1.3%)		5.12 (3.28–8.13)	<0.001
Black African	118 (1.1%)	72 (6.0%)	113 (4.2%)		4.63 (3.67–5.87)	<0.001
Other ethnic group	1,088 (9.9%)	131 (11%)	212 (7.9%)		0.93 (0.82–1.06)	0.3
Smoking				0.7		
Non-smoker	9,587 (87%)	1,037 (86%)	2,345 (88%)		–	–
Ex-smoker	900 (8.2%)	107 (8.9%)	219 (8.2%)		1.03 (0.90–1.17)	0.7
Light smoker (<10 cigarettes/day)	283 (2.6%)	33 (2.8%)	55 (2.1%)		0.88 (0.69–1.12)	0.3
Moderate smoker (10–19 cigarettes/day)	154 (1.4%)	13 (1.1%)	38 (1.4%)		0.94 (0.68–1.28)	0.7
Heavy smoker (>=20 cigarettes/day)	55 (0.5%)	9 (0.8%)	13 (0.5%)		1.13 (0.68–1.83)	0.6
Systolic BP (mmHg)	125 (115, 134)	125 (115, 134)	125 (116, 135)	0.3	1 (1.00–1.00)	0.5
BMI (kg/m²)	25.1 (22.6, 28.2)	25.3 (22.6, 28.3)	25.5 (23.1, 28.5)	<0.001	1.01 (1.01–1.02)	<0.001
Diabetes	147 (1.3%)	12 (1.0%)	43 (1.6%)	0.3	1.06 (0.77–1.44)	0.7
Medical history	605 (5.5%)	61 (5.1%)	162 (6.1%)	0.4	1.05 (0.89–1.23)	0.6
Migraine	250 (2.3%)	25 (2.1%)	53 (2.0%)	0.6	0.88 (0.68–1.14)	0.3
Family history CVD	2,225 (20%)	248 (21%)	622 (23%)	0.003	1.14 (1.04–1.25)	0.003
Medication	470 (4.3%)	43 (3.6%)	218 (8.2%)	<0.001	1.62 (1.38–1.89)	<0.001

Values are presented as median (Q1, Q3) or *n* (% of the total study population within Lp(a) grouping). To avoid potential for re-identification factors (where numbers were <5) certain factors were grouped together: Chinese ethnicity was grouped with other ethnic group, diabetes includes type 1 and type 2; medical history included chronic kidney disease, rheumatoid arthritis, systemic lupus erythematosus, erectile dysfunction and severe mental illness; medications include statins, steroid tablets and atypical antipsychotic medication. Odds ratios (OR), 95% confidence intervals (CI), and *p*-values calculated via univariable logistic regression using Lp(a) risk grouping as the dependent variable.

a Median (Q1, Q3); *n* (%).

b Kruskal–Wallis rank sum test; Pearson's Chi-squared test.

c Odds ratios (OR), 95% confidence intervals (CI), and *p*-values calculated via univariable logistic regression using LP(a) risk grouping as the dependent variable.

### Characteristics associated with elevated Lp(a)

Compared with participants having Lp(a) ≤125 nmol/L, those with elevated Lp(a) were older (43 vs. 41 years, *p* < 0.001); had higher BMI (25.5 vs. 25.1 kg/m^2^, *p* < 0.001); higher median QRISK3 scores (1.8% vs. 1.4%, *p* < 0.001); more frequently reported a family history of CVD (23% (622/2,670) vs. 20% (2,473/12,178), *p* < 0.001); and were more likely to be prescribed statins (8.1% (215/2,670) vs. 3.9% (481/12,178), *p* < 0.001). No significant associations were observed for smoking status, systolic blood pressure, diabetes, atrial fibrillation, chronic kidney disease, rheumatoid arthritis, migraine, or severe mental illness. Ethnic variation was observed, with relatively higher proportions of Black African and Black Caribbean participants among the high risk Lp(a) groups (Table 1).

### Relationship between elevated Lp(a) and QRISK3

Among participants included in the analysis, 9.6% (1,426/14,848) were classified as having an estimated QRISK3 score ≥10%, while 90.4% (13,422/14,848) were classified as having a QRISK3 score <10%. Distribution across the three Lp(a) categories was: low risk Lp(a) (≤75 nmol/L), 74% (10,979/14,848); grey-zone Lp(a) (76–125 nmol/L), 8.1% (1,199/14,848); and high risk Lp(a) (>125 nmol/L),18% (2,670/14,848) (Table 2).

**Table 2:** Association between Lp(a) risk categories and estimated QRISK3 cardiovascular risk.

	Lp(a) groups			
QRISK3 groups	Low risk	Grey zone	High risk	Total
Low risk	9,966 (67%)	1,085 (7.3%)	2,371 (16%)	13,422 (90%)
High risk	1,013 (6.8%)	114 (0.8%)	299 (2.0%)	1,426 (9.6%)
Total	10,979 (74%)	1,199 (8.1%)	2,670 (18%)	14,848 (100%)

Values are presented as *n* (% of the total study population). Participants were categorised according to Lp(a) concentration into low risk (≤75 nmol/L), grey zone (76–125 nmol/L), and high risk (>125 nmol/L) groups, and according to estimated QRISK3 risk as low risk (<10%) or high risk (≥10%).

Although elevated Lp(a) concentrations were more common among participants with QRISK3 scores ≥10%, overall concordance between the two risk stratification approaches was limited. Of individuals with elevated Lp(a) concentrations, 11% (299/2,670) also had a QRISK3 score ≥10%, while 89% (2,371/2,670) remained below the conventional QRISK3 treatment threshold (Table 2). Consequently, 16.0% (2,371/14,848) of the entire study population exhibited elevated Lp(a) levels despite being categorised as low cardiovascular risk, according to estimated QRISK3.

Similarly, the grey-zone Lp(a) group demonstrated substantial discordance with QRISK3 risk classification. Among participants with Lp(a) concentrations between 76 and 125 nmol/L, 90.5% (1,085/1,199) had a QRISK3 score <10%, while only 9.5% (114/1,199) had a QRISK3 score ≥10% (Table 2). Together, the grey-zone and high risk Lp(a) groups accounted for 23.3% (3,456/14,848) of the cohort and were classified as low risk by QRISK3 despite evidence of intermediate or elevated inherited cardiovascular risk based on Lp(a) concentrations.

Across Lp(a) categories, median QRISK3 scores increased progressively from 1.4% (IQR 0.4–4.2) in the low risk Lp(a) group to 1.4% (IQR 0.5–4.6) in the grey-zone group and 1.8% (IQR 0.6–5.1) in the high risk Lp(a) group (*p* < 0.001) (Table 2). However, the absolute differences were modest, indicating that elevated Lp(a) frequently occurs independently of the traditional cardiovascular risk factors that contribute to QRISK3 estimation.

## Discussion

This study demonstrates that elevated Lp(a) is common within a large contemporary UK health-screening population and that concordance between elevated Lp(a) levels and elevated estimated QRISK3 risk is relatively limited. Although participants with higher Lp(a) concentrations exhibited modestly higher QRISK3 scores, the overlap between these two approaches to risk assessment was considerably less than might be expected if both were capturing the parameters of cardiovascular risk (3, 4, 10).

The most clinically important finding was that almost nine out of ten individuals (89%, 2,371/2,670) with elevated Lp(a) concentrations (>125 nmol/L) had QRISK3 scores below 10%, while 16% (2,371/14,848) of the entire cohort exhibited elevated Lp(a) despite being classified as low cardiovascular risk according to QRISK3. These observations demonstrate substantial discordance between Lp(a)-based risk stratification and QRISK3 risk categorisation. While the present study cannot determine whether these individuals will experience higher future cardiovascular event rates, it highlights that elevated Lp(a), a recognised cardiovascular risk factor, is frequently present among individuals classified as low risk by conventional risk algorithms.

A key explanation for this discordance is that QRISK3 and Lp(a) describe different but complementary dimensions of cardiovascular risk. QRISK3 is heavily influenced by age and the cumulative burden of traditional risk factors and is designed to estimate the probability of a cardiovascular event within the next decade. In contrast, Lp(a) represents a genetically determined trait that is present from birth and has been associated with lifetime cardiovascular risk (12–14). Consequently, elevated Lp(a) can be present in otherwise healthy younger adults whose 10-year cardiovascular risk remains low despite the presence of an established inherited risk factor.

An important and often overlooked finding from this study relates to the subgroup of individuals with Lp(a) concentrations between 76 and 125 nmol/L. While concentrations above 125 nmol/L are increasingly recognised as clinically significant, the management of individuals within the intermediate or “grey-zone” range remains less clearly defined (4, 10). In the present cohort, more than one thousand individuals fell into this category, representing over 8% (1,199/14,848) of all participants. Similar to those with markedly elevated Lp(a)most individuals within this grey-zone range were classified as low risk, according to QRISK3. This finding further illustrates the limited overlap between Lp(a)-based categorisation and conventional short-term risk assessment.

The concept of a grey zone is particularly important because the relationship between Lp(a) concentration and cardiovascular risk appears continuous rather than threshold dependent. Although individuals with grey-zone concentrations may not warrant the same degree of intervention as those with markedly elevated Lp(a) levels, they may nevertheless represent a group in whom cardiovascular risk is incrementally increased. Consequently, the knowledge of grey-zone Lp(a) concentrations may contribute to a broader assessment of cardiovascular risk, particularly among individuals with additional risk-enhancing features such as a family history of premature cardiovascular disease, elevated LDL-cholesterol, metabolic dysfunction, or QRISK3 estimates approaching treatment thresholds.

From a clinical perspective, grey-zone Lp(a) concentrations may be best viewed as a risk-enhancing factor rather than a stand-alone indication for treatment (15). In this context, knowledge of Lp(a) status can help refine interpretation of QRISK3 estimates and support a more personalised approach to primary prevention (16). Such an approach aligns with contemporary precision-medicine models in which genetic, biochemical and conventional risk factors are considered collectively rather than independently.

The findings therefore support the emerging view that Lp(a) should not be considered a replacement for established risk prediction tools, but rather a risk-enhancing factor that provides information not captured by traditional cardiovascular risk algorithms (17, 18). This concept is particularly relevant for individuals whose QRISK3 scores are close to treatment thresholds and for those with elevated or markedly elevated Lp(a) concentrations despite otherwise favourable risk profiles. In such circumstances, knowledge of Lp(a) status may support clinical decision making regarding risk factor modification, intensity of preventive measures, and consideration of specialist referral where appropriate.

Rather than interpreting QRISK3 and Lp(a) independently, a combined approach enables identification of distinct clinical phenotypes. Individuals with both high QRISK3 and elevated Lp(a) represent a group with a greater burden of recognised cardiovascular risk factors who may warrant particularly intensive preventative management (Table 3).

**Table 3:** Conceptual framework illustrating the relationship between QRISK3 risk categories and Lp(a) categories.

QRISK3 groups	Lp(a) Low Risk(≤75 nmol/L)	Lp(a) Grey Zone (76–125 nmol/L)	Lp(a) High Risk (>125 nmol/L)
QRISK3 Low Risk (<10%)	Conventional and Lp(a)-based assessment concordant.	Low estimated 10-year risk with presence of an intermediate Lp(a)-risk	Low estimated 10-year risk despite the presence of a recognised inherited cardiovascular risk factor.
QRISK3 High Risk (≥10%)	Elevated estimated risk primarily driven by conventional QRISK3 factors.	Elevated estimated risk with additional Lp(a)-related risk information.	Elevated estimated risk with coexisting conventional and Lp(a)-associated cardiovascular risk factors.

Conversely, individuals with low QRISK3 but elevated Lp(a) represent a population in whom a recognised inherited cardiovascular risk factor is present despite low estimated 10-year risk. While the present study cannot determine whether such individuals experience a higher observed event rate than predicted by QRISK3, it demonstrates that Lp(a) provides information that is not reflected in conventional risk scores. This combined framework aligns with the growing recognition that precision prevention requires consideration of both traditional and genetically mediated risk factors.

The integration of this three-tier approach with QRISK3 may provide a more nuanced framework for cardiovascular risk assessment by incorporating both conventional risk factors and inherited cardiovascular susceptibility. However, prospective outcome studies are required to determine whether this approach improves cardiovascular risk prediction or clinical outcomes.

The table illustrates how QRISK3 and Lp(a) may identify different dimensions of cardiovascular risk. QRISK3 reflects estimated 10-year cardiovascular risk based on conventional risk factors, whereas Lp(a) reflects a genetically determined cardiovascular risk factor. The framework is intended to aid interpretation of the observed discordance between the two approaches and should not be interpreted as a management algorithm. Prospective studies incorporating cardiovascular outcomes are required to determine whether combining QRISK3 and Lp(a) improves risk prediction or clinical decision-making.

The observed ethnic variation in Lp(a) concentrations, particularly among Black African and Black Caribbean participants, is consistent with previous epidemiological studies and reinforces the value of direct measurement rather than reliance on conventional risk-factor assessment alone. Importantly, these findings further support recommendations for at least one-time Lp(a) testing across diverse populations (19, 20).

Collectively, these findings support a three-tier interpretation of Lp(a) rather than a simple normal-vs.-abnormal framework. Individuals with low Lp(a) appear adequately characterised by conventional risk assessment; those with markedly elevated Lp(a) represent a distinct inherited high risk phenotype; and those within the grey zone occupy an intermediate position where Lp(a) may act as a clinically useful risk-enhancing factor. The integration of this three-tier approach with QRISK3 may provide a more nuanced and clinically actionable framework for cardiovascular risk stratification than either measure alone. Furthermore, the present findings should also be considered within the broader context of longitudinal studies evaluating Lp(a) and cardiovascular outcomes. Large prospective cohorts, including the Framingham Heart Study and subsequent population-based investigations, have consistently demonstrated that elevated Lp(a) is associated with increased risk of atherosclerotic cardiovascular disease independent of conventional risk factors (4, 13, 21, 22). These observations support the concept that Lp(a) provides prognostic information that may not be fully captured by contemporary multivariable risk scores and may be particularly relevant when considering lifetime rather than short-term cardiovascular risk.

Our findings may also have implications for future health economics and outcomes research. The identification of a substantial subgroup of individuals with elevated Lp(a) despite low estimated QRISK3 risk raises important questions regarding the potential clinical and economic value of broader Lp(a) testing, earlier identification of inherited cardiovascular risk, and targeted preventive interventions. Modelling studies incorporating incident cardiovascular events, healthcare utilisation, and emerging Lp(a)-lowering therapies could help determine the cost-effectiveness of incorporating routine Lp(a) measurement into cardiovascular prevention pathways.

Furthermore, the current study highlights opportunities for future validation using large longitudinal electronic health record resources such as TriNetX (23). Recently, Mahmoud et al. utilised the TriNetX network to evaluate more than 24,000 individuals with elevated Lp(a) and demonstrated favourable cardiovascular outcomes among patients receiving GLP-1 receptor agonist therapy (24). The accompanying editorial highlighted the ability of large-scale real-world datasets to generate clinically relevant evidence in populations defined by elevated Lp(a). Such platforms could be used to examine cardiovascular outcomes among individuals with discordant QRISK3 and Lp(a) profiles, determine whether elevated Lp(a) confers additional risk beyond conventional risk scores, and assess the clinical utility of incorporating Lp(a) into future risk prediction frameworks.

## Limitations

Several limitations of this study should be acknowledged when interpreting the findings. First, QRISK3 scores were estimated from the available dataset rather than generated directly using a validated clinical QRISK3 calculation platform. Although the variables required for QRISK3 risk estimation were comprehensively captured, the absence of the official calculator may have introduced a degree of measurement error and could have resulted in minor differences from clinically derived risk scores.

Second, some components of the QRISK3 algorithm were unavailable. Specifically, the Townsend deprivation score, an important measure of socioeconomic deprivation incorporated into QRISK3, could not be included in the risk calculations. Given the established association between socioeconomic status and cardiovascular risk, omission of this variable may have led to under- or overestimation of risk in certain individuals. Additionally, blood pressure variability, another variable included in the QRISK3 algorithm, was not available and therefore could not be accounted for in the risk estimates.

Third, the dataset did not include any participants classified as Chinese ethnicity. As QRISK3 incorporates ethnicity-specific risk adjustments, the absence of this population limits the applicability of the findings to Chinese individuals and reduces the ethnic diversity represented within the study cohort.

Fourth, the cross-sectional nature of the analysis restricts interpretation to associations observed at a single point in time. Consequently, it is not possible to determine causal relationships between Lp(a) concentrations and cardiovascular risk, nor to assess the predictive value of elevated Lp(a) for future cardiovascular events. Longitudinal follow-up studies are required to evaluate incident cardiovascular outcomes and determine the extent to which Lp(a) provides incremental prognostic information beyond established risk prediction tools.

Finally, the study population consisted of individuals who voluntarily attended health screening assessments. This may have introduced selection bias, as participants may differ from the general UK population in terms of health awareness, socioeconomic status, health-seeking behaviours, and underlying cardiovascular risk profiles. A further limitation is that participants were not excluded based on established cardiovascular disease, prior myocardial infarction, stroke, peripheral arterial disease, or coronary revascularisation. The cohort should therefore be interpreted as a real-world health-screening population rather than a strictly defined primary-prevention population, and future sensitivity analyses excluding individuals with established cardiovascular disease would be valuable to determine whether the observed discordance between Lp(a) classification and QRISK3 risk persists in a primary-prevention subgroup. Therefore, the findings may not be fully generalisable to the wider population.

Despite these limitations, the large sample size, real-world clinical setting, comprehensive capture of major QRISK3 variables, and detailed evaluation of clinically relevant Lp(a) thresholds provide valuable insights into the relationship between Lp(a) concentrations and estimated cardiovascular risk within a contemporary UK health-screening cohort.

## Conclusions

In this large real-world UK health screening cohort of almost 15,000 participants, elevated Lp(a) concentrations were common, affecting approximately 18% of individuals. In contrast, only 9.6% of participants were classified as having elevated cardiovascular risk according to an estimated QRISK3 score of ≥10%. These findings highlight a substantial discrepancy between Lp(a)-based risk identification and conventional cardiovascular risk assessment using QRISK3.

Notably, most individuals with elevated Lp(a) did not meet established QRISK3 thresholds for increased cardiovascular risk. This limited overlap suggests that Lp(a) captures an additional dimension of cardiovascular risk that may not be adequately reflected by traditional multivariable risk prediction models. Given that Lp(a) is a largely genetically determined risk factor associated with lifelong exposure to atherogenic particles, individuals with elevated levels may have increased lifetime cardiovascular risk despite having a low estimated short- to intermediate-term risk according to conventional risk calculators.

These findings support the concept that Lp(a) and QRISK3 provide complementary rather than interchangeable information for cardiovascular risk assessment. Reliance on traditional risk prediction tools alone may not identify a substantial proportion of individuals with elevated inherited cardiovascular risk associated with high Lp(a) concentrations.

Routine incorporation of Lp(a) measurement alongside established cardiovascular risk assessment approaches has the potential to improve risk stratification by identifying individuals who may otherwise be classified as low risk by conventional risk scores. Earlier recognition of elevated Lp(a) may facilitate more personalised preventive strategies, including optimisation of modifiable risk factors, consideration of more intensive lipid-lowering approaches when appropriate, and targeted clinical follow-up. As recommendations for Lp(a) testing become increasingly integrated into clinical practice, the inclusion of Lp(a) within cardiovascular prevention pathways may help address an important gap in current risk assessment frameworks.

## Data Availability

The raw data supporting the conclusions of this article will be made available by the authors, without undue reservation.
